# Optimized pediatric emergency nursing and Its effects on successful resuscitation and adverse reactions in children

**DOI:** 10.3389/fped.2025.1578177

**Published:** 2025-06-03

**Authors:** Xia Zhang, Xiao Chen

**Affiliations:** Xiangyang Central Hospital, Affiliated Hospital of Hubei University of Arts and Science, Xiangyang City, Hubei Province, China

**Keywords:** pediatric emergency care, nursing interventions, successful resuscitation, adverse effects, satisfaction, stable vital signs

## Abstract

**Objective:**

To evaluate impacts of optimized pediatric emergency care on successful resuscitation and adverse events of children in the emergency department.

**Methods:**

Pediatric patients who visited our hospital's emergency room between January 2022 and December 2023 were chosen as the study participants. These patients were randomly divided into the study group (using optimized emergency care model) and the control group (using conventional emergency care model). The two groups were compared for the rate of successful resuscitation, stabilization time of vital signs, average length of hospital stay, incidence of adverse reactions, parental satisfaction with nursing care, scores of Self-Assessment Scale of Anxiety (SAS) and Self-Assessment Scale of Depression (SDS) before and after nursing care, and assessment of life quality.

**Results:**

A total of 140 children were included in this study. Seventy patients each were assigned to study and control groups. After the intervention, the study group's successful resuscitation rate was considerably greater than the control's (*P* < 0.05). For the study group, the optimized care intervention significantly reduced the stabilization time of vital signs and the typical duration of hospitalization compared with the control group (*P* < 0.001). Compared to the control group, the study group had a lower incidence of adverse reactions (*P* < 0.05), a higher level of parental satisfaction (*P* < 0.05), significant reduction of the SAS and SDS scores (*P* < 0.001), and significant elevation of the quality of life scores (*P* < 0.001).

**Conclusion:**

The study demonstrates that the optimized pediatric emergency care intervention is an effective approach for improving the successful resuscitation of children in emergency medicine, and mitigate the incidence of adverse reactions. Concurrently, the optimized nursing intervention was found beneficial for anxiety and depression levels, with a notable improvement in their quality of life as well as parental satisfaction. Optimized nursing interventions therefore are valuable and are recommended for wider pediatric emergency care practice.

## Introduction

In today's healthcare system, the quality of pediatric emergency services is directly related to the survival of pediatric patients and their subsequent quality of life (1). Globally, pediatric emergency services face numerous challenges, including: (1) variability in the availability of specialized equipment. For example, in low-income regions, the scarcity of functional ventilators has led to hundreds of thousands of preventable pediatric deaths due to lower respiratory tract infection (2). It could also be variability caused by equipment failure or improper operation of medical equipment by nurses. (2) Disparities in training levels of healthcare providers. A national assessment in the United States shows that only 70% of pediatric emergency departments have procedures to notify healthcare providers when vital signs are abnormal (3). Excessive workload or insufficient training of emergency nurses may be the reasons for lacking vital sign recordings and notifications. (3) Inconsistent adherence to standardized emergency protocols. In the process of dealing with pediatric cardiac arrest, deviation from evidence-based guidelines on pediatric advanced life support have been frequently reported, which are related to lower rate of successful return of spontaneous circulation (4). With advances in medical technology and clinical practice, it is particularly important to optimize pediatric emergency care interventions (5, 6). The traditional model of emergency care, although reliable in some circumstances, may not fully meet the emergency care needs of pediatric patients and their families (7, 8). Children's physiological and psychological needs are distinctly different from those of adults, demanding specific approaches in emergency care. For instance, their smaller airways and higher metabolic rates require rapid and precise intervention, while their psychological vulnerability demands sensitive communication and emotional support (9).

This current state of affairs has prompted healthcare professionals to explore new approaches to care in order to improve resuscitation success and overall treatment outcomes while reducing the incidence of adverse events (10–13). Studies to date have shown improvements in quality of emergency care can significantly affect treatment outcomes (14). Specifically, optimized care interventions not only improve the child's chances of survival, but also improve his or her mental health status and quality of life, which are key indicators for assessing the effectiveness of emergency care (9, 15, 16). In addition, the assessment of parental satisfaction with care serves as a crucial element in the evaluation of emergency care quality, as high satisfaction is usually closely associated with better treatment adherence and outcomes (16, 17).

Currently, an innovative and optimized pediatric emergency care program is scarce and necessary to address the challenges faced by pediatric emergency medical services. To improve the quality of pediatric emergency services and explore the effects of optimized pediatric emergency care in our hospital, our study intended to mainly evaluate successful resuscitation rate and the incidence of adverse events after implementing optimized emergency care interventions in children. We expected to provide a scientific improvement plan for pediatric emergency care, which can be replicated nationwide to improve overall emergency medical service quality and efficiency.

## Materials and methods

### Study design

From January 1, 2022, to December 31, 2023, pediatric patients who visited our hospital's emergency department participated in this study. Patients were randomly assigned to the study group and the control group. An optimized emergency care intervention model was administered throughout to the group in the study, while a control group was administered the conventional emergency care model.

Inclusion criteria: (1) The patients involved in this study were children under 14 years of age admitted to pediatric emergency care with acute and critical illnesses; (2) Complete clinical records of the children were available. Exclusion criteria: (1) Neonates who were directly transferred from the delivery room to the emergency department within 1 day of birth; (2) Children with cardiac arrest were excluded from this emergency study.

The informed consent form was duly signed by the children's families. This study adopted the deferred consent measure. Necessary first aid measures were implemented first. After the child's condition stabilized (usually within 24–72 h after the first aid measures), medical staff explained to the guardians and requested them to sign the consent form (to continue participation or withdraw).

### Intervention measures

Interdisciplinary collaboration among emergency doctors, nurses, psychologists and social workers provided the overall emergency care plan. The control group adopted the standard pediatric emergency care process according to the guidelines for care of children in the emergency department, including immediate assessment and initiation of necessary care measures for the children received, such as monitoring heart rate, blood pressure and performing ECG monitoring, and other routine care operations, which were performed strictly in accordance with the instructions provided by the doctor; nurses should receive training in skills and knowledge related to emergency care for children of all ages; ensuring that first aid equipment and supplies are placed in the first aid room; and arranging a nurse coordinator for pediatric emergency (18). In the study group, on the basis of the standard care, some optimized emergency care interventions were implemented, including: (1) Nursing team capacity enhancement: to ensure that all emergency nursing staff not only possessed basic nursing skills, but also possessed advanced nursing experience and professional knowledge to handle special situations, such as advanced life support (ECMO, POCUS). Regular training and assessment of these skills for medical and nursing staff, only those who pass the assessment can participate in first aid work. (2) Resuscitation equipment and drug management: eestablish electronic archive for equipment, record information such as model, purchase time, warranty period, and maintenance records, and update them in real time through the equipment management system. Connect to the hospital HIS system to record the drug inventory in real time. Based on the disease data of emergency pediatric visits in the past year, optimize the reserve of specialized drugs for children. (3) Optimization of nursing process and emergency management: establish a green channel for pediatric emergencies (such as trauma, sepsis, arrhythmia). Apply the electronic medical record and intelligent triage system to synchronize the data of patients to the medical staff workstations in real time, and streamline the format and method of nursing records to improve work efficiency. (4) Family communication and psychological support: Experienced nursing staff are responsible for explaining the necessity and importance of nursing measures to children's families, improving cooperation, and optimizing the overall nursing experience. Allow family members to participate in the care process of the child patients, providing psychological support and health education.

### Observation indicators

(1) Successful resuscitation rate (the primary outcome): the proportion of critically ill or injured children who survived and achieved the predetermined physiological, functional or developmental endpoints after emergency resuscitation intervention within 10 days after hospitalization. For example, for children with cardiac arrest, the successful resuscitation rate was the proportion of pediatric cardiac arrest patients who achieved return of spontaneous circulation (ROSC) (19); (2) Vital signs stabilization time: the time taken to assess the vital signs of the patients (e.g., heart rate and blood pressure) to reach a state without significant fluctuations, and the stabilization of vital signs is based on the absence of large fluctuations in heart rate, blood pressure, etc.; (3) Mean duration of hospitalization: the average number of days from admission to discharge, calculated as the sum of the number of days of hospitalization of all children/ the total number of children; (4) Incidence of adverse reactions: relating to the equipment and relevant personnel (such as equipment failure, improper operation, insufficient equipment disinfection, and insufficient battery power of the monitoring equipment), relating to the transfer process (such as delay during the transfer process, improper transferring operation, lack of effective monitoring of the children's conditions during the transfer process), and relating to the illness (such as aggravation of the patient's condition, occurrence of complications, drug reaction, excessive high or low body temperature, and multiple organ dysfunction); (5) This study used a self-developed rating scale referring to other existing scales, scored from 0 to 100, to assess parental satisfaction with care (20–23). Scores over 90 were considered “very satisfied”, a score of 60–89 as “satisfied”, and a score of less than 60 as “dissatisfied”. The formula employed for determining overall satisfaction with nursing care was: total satisfaction with nursing care = (number of very satisfied cases + number of satisfied cases)/total sample size × 100%; (6) The emotional state of the pediatric patients was evaluated using the Self-Assessment Scale of Anxiety (SAS) and Self-Assessment Scale of Depression (SDS) (20, 21). The SAS assesses the anxiety level of patients, with scores ranging from 0 to 100 and a cut-off value of 50, where 50–59 is considered mild anxiety, 60–69 is considered moderate anxiety and over 70 is considered severe anxiety. The SDS assesses the degree of depression in patients on a scale from 0 to 100. The cut-off value was 53 points, with 53–62 being mild depression, 63–72 being moderate depression, and over 73 being severe depression; (7) Quality of life was assessed with the use of the five subscales of the Short Form 36 Health Survey (SF-36) (22, 23): physical functioning, social functioning, role functioning, cognitive functioning, and general health. The total score for each item is 100 points; the higher the score, the more perfect it is according to the corresponding function.

### Study size

The primary outcome in this study was the successful resuscitation rate. We formulated the hypotheses as follows: the study group with optimized emergency care will have higher successful resuscitation rate; the control group with standard emergency care will have higher successful resuscitation rate. Considering *α* = 0.05, *Uα* = 1.96, *β =* 0.2, *Uβ =* 0.8416, successful resuscitation rate in the study group (*T*) = 0.9429, successful resuscitation rate in the control group (*C*) = 0.7571, study group: control group = 1: 1, N1 was the sample size in the study group, N2 was the sample size in the control group, the number of subjects required to demonstrate a significant difference of successful resuscitation rate between the study group and the control group was 55 patients. The calculation formula was as follows:

$$N1=N2={\left[\frac{\left(U\alpha +U\beta \right)}{\left|T-C\right|}\right]}^{2}\times [T(1-T)+C(1-C)]$$

Considering a loss to follow-up rate of 20%, each of the study group and the control group required 62 samples, and a minimum sample size of 122 cases was required in total.

### Statistical methods

The data in this study were analyzed using SPSS 26 software. Categorical variables were described as frequencies and percentages [*n* (%)]. Continuous variables that adhered to a normal distribution were reported as mean ± standard deviation $\left(\bar{x}\pm s\right)$, while those that did not were reported as median with interquartile range. Numbers (percentages) were used to represent categorical variables. Intergroup comparisons for continuous variables were made using paired *t*-tests or ANOVA for normally distributed data, and Mann–Whitney *U* test or Kruskal–Wallis test for non-normally distributed data. The comparison of categorical variables between groups was performed using the chi-square test (*χ*^2^ test). The criterion for statistical significance was a *p*-value of under 0.05.

## Results

### Comparative analysis of clinical information in both groups

After inclusion and exclusion criteria, a total of 140 pediatric patients were included in this study. According to a ratio of 1:1, the patients were randomly divided into the study group and the control group, with 70 people in each group. The general clinical data, such as age and gender, of the two groups were compared, and the statistical results show no significant difference existed between two groups (*P* > 0.05), indicating balanced baseline information between groups and the study results were comparable (Table 1).

**Table 1:** Comparison of general clinical data between the two groups.

General clinical data	Control group (*n* = 70)	Study group (*n* = 70)	*t*/*χ*^2^	*P*
Gender [*n* (%)]				
Male	34 (48.57%)	32 (45.71%)	0.115	0.735
Female	36 (51.43%)	38 (54.29%)		
Age (years, $\bar{x}\pm s$)	6.75 ± 2.54	6.27 ± 3.02	1.102	0.275

### Comparison of resuscitation success rate, vital signs stabilization time, and length of hospital stay

Table 2 indicates that the successful resuscitation rate in the study group was significantly higher compared to the control group after interventions (*P* = 0.002). Meanwhile, the vital signs stabilization time and the average length of hospital stay were both significantly shorter in the study group than in the control group (*P* < 0.001).

**Table 2:** Comparison of successful resuscitation rate, vital signs stabilization time and average length of hospital stay.

Group	Successful resuscitation rate [*n* (%)]	Vital signs stabilization time (*d*, $\bar{x}\pm s$)	Average length of hospital stay (*d*, $\bar{x}\pm s$)
Control group (*n* = 70)	53 (75.71%)	2.23 ± 0.42	8.25 ± 1.35
Study group (*n* = 70)	66 (94.29%)	1.80 ± 0.54	6.13 ± 1.03
*t*/*χ*^2^	9.468	4.804	9.720
*P*	0.002	<0.001	<0.001

### Assessment of the incidence of adverse reactions

In Table 3, the assessment of the incidence of adverse reactions revealed that the study group had a significantly lower total incidence of adverse reactions (including adverse reactions relating to the equipment and relevant personnel, the transfer process, and the illness) compared to the control group (*P* = 0.024).

**Table 3:** Assessment of the incidence of adverse reactions.

Group	Relevant to equipment and personnel [*n* (%)]	Relevant to transfer process [*n* (%)]	Relevance to the illness [*n* (%)]	Total incidence [*n* (%)]
Control group (*n* = 70)	4 (5.71%)	3 (4.29%)	4 (5.71%)	11 (15.71%)
Study group (*n* = 70)	1 (1.43%)	1 (1.43%)	1 (1.43%)	3 (4.29%)
*χ* ^2^				5.079
*P*				0.024

### Assessment of parents' satisfaction with nursing care

In Table 4, the survey on parental satisfaction indicates that parents in the study group were significantly more satisfied (including very satisfied and satisfied) with the care their children received compared to parents in the control group (*P* = 0.014).

**Table 4:** Parents’ assessment of satisfaction with care.

Group	Very satisfied [*n* (%)]	Satisfied [*n* (%)]	Unsatisfied [*n* (%)]	Total satisfied [*n* (%)]
Control group (*n* = 70)	25 (35.71%)	33 (47.14%)	12 (17.14%)	58 (82.86%)
Study group (*n* = 70)	37 (52.86%)	30 (42.86%)	3 (4.29%)	67 (95.71%)
*χ* ^2^				6.048
*P*				0.014

### Comparison of SAS and SDS scores between groups before and after interventions

In Table 5, analysis of pre- and post-intervention SAS and SDS scores shows a significant reduction in anxiety and depression levels in the study group (*P* < 0.001).

**Table 5:** Comparison of SAS and SDS scores before and after interventions in the two groups.

Group	SAS (point, $\bar{x}\pm s$)		SDS (point, $\bar{x}\pm s$)	
	Before	After	Before	After
Control group (*n* = 70)	61.39 ± 3.53	53.03 ± 2.88	63.57 ± 5.92	52.71 ± 3.49
Study group (*n* = 70)	60.80 ± 4.74	42.37 ± 4.51	62.03 ± 3.74	40.44 ± 3.97
*t*	0.751	14.925	1.733	17.685
*P*	0.454	<0.001	0.086	<0.001

### Comparison of quality of life scores between the groups

Using SF-36 to assess the children's quality of life, it was found in Table 6 that the study group had significantly higher scores (including physical functioning, social functioning, role functioning, cognitive functioning, and general health) than the control group after the intervention (*P* < 0.001).

**Table 6:** Comparison of quality of life scores between the two groups before and after interventions.

Group	Physical functioning (point, $\bar{x}\pm s$)		Social functioning (point, $\bar{x}\pm s$)		Role functioning (point, $\bar{x}\pm s$)		Cognitive functioning (point, $\bar{x}\pm s$)		General health (point, $\bar{x}\pm s$)	
	Before	After	Before	After	Before	After	Before	After	Before	After
Control group (*n* = 70)	73.32 ± 7.32	82.40 ± 5.65	60.42 ± 6.86	83.63 ± 7.69	67.22 ± 9.29	80.26 ± 6.56	57.68 ± 10.70	72.51 ± 6.42	66.43 ± 7.66	75.01 ± 7.15
Study group (*n* = 70)	74.47 ± 6.59	90.38 ± 7.92	61.93 ± 5.99	91.43 ± 7.85	66.75 ± 6.13	86.44 ± 7.62	56.18 ± 8.50	89.64 ± 8.15	65.15 ± 8.21	88.87 ± 8.38
*t*	−0.904	−6.179	−1.280	−5.438	0.328	−4.679	0.855	−12.499	0.871	−9.562
*P*	0.368	<0.001	0.203	<0.001	0.743	<0.001	0.394	<0.001	0.386	<0.001

## Discussion

Pediatric emergency healthcare services face many challenges, particularly how to effectively respond to acute conditions and provide timely medical interventions (24, 25). With advances in medical technology and increasing patient needs, traditional models of emergency care may no longer be sufficient to meet current demands for quality and efficiency in healthcare (26, 27). In emergency situations, pediatric patients in particular require a more refined and personalised approach to care due to their unique physiological and psychological needs (28). This study aimed to evaluate the effectiveness of a new optimized pediatric emergency care intervention model. By comparing the difference in outcomes between the optimized care model and the traditional emergency care model in the management of emergency pediatric patients, this study sought to verify whether the optimized care intervention was effective in increasing the success rate of rescue, decreasing the incidence of adverse events, and exploring its potential impact on children's mental health and quality of life.

The findings showed that the optimization of care measures significantly improved the successful resuscitation rate, which could be attributed to a more systematic and professional care process and efficient execution of first aid skills by nursing staff. The notable increase in the successful resuscitation rate directly translates to a higher chance of survival for critically ill pediatric patients. A systematic care process and proficient execution of first aid skills help minimize the risk of irreversible organ damage and improve long-term prognosis. Significant reductions in the vital signs stabilization time and length of hospital stay reflected the improved treatment efficiency and nursing responsiveness of the optimized measures, which not only improves the utilization efficiency of hospital resources but also reduces the economic burden on families. In addition, the incidence of adverse events was lower in the study group than in controls, which indicates that the optimization measures were effective in reducing risks and complications during medical treatment. Fewer adverse events not only reduce the physical suffering of children but also lower the risk of secondary health problems. Optimization of nursing interventions significantly increased parental satisfaction with nursing care, which may be due to improved quality of nursing services and increased communication between nursing staff and parents. The boost in parental satisfaction has a positive impact on the patient-family-healthcare provider relationship, and helps improve the compliance of pediatric patients with treatment plans. Regarding mental health indicators, children in the study group scored significantly lower on the SAS and SDS for anxiety and depression compared to the control group, reflecting that the optimization measures helped to reduce the children's psychological stress. Meanwhile, the improvement in quality of life scores further validated the positive impact of optimized care. These positive impacts promote the emotional well-being and healthy growth of pediatric patients.

Some optimized and innovative emergency care models have proven their positive impacts on the treatment outcomes of patients. For example, the inpatients in the emergency department using a prospective information-based nursing model had considerably lower rate of adverse events, shouter emergency response time, and higher nursing compliance rate than those using routine nursing mode (29). Additionally, the positive effects of graded emergency nursing model has been validated in multiple studies, reflected in shortening waiting and triage times, enhancing resuscitation success rates, reducing complication incidence, and improving patient prognosis and nursing satisfaction (30, 31). The optimized emergency care model of this study is also a new opportunity for pediatric patients, which is expected to bring positive impacts to more pediatric patients.

This study offers innovative insights into pediatric emergency care by systematically implementing comprehensive improvements, unlike previous studies that focused on adult care optimization or single interventions. It demonstrates real-world benefits such as increased successful resuscitation and reduced adverse events, while also addressing the psychological health and quality of life of children and their families, filling a research gap. The optimized interventions significantly improved successful resuscitation rates, sped up vital sign stabilization, shortened hospital stays, reduced adverse events, and enhanced parental satisfaction and children's quality of life. These findings suggest important applications for optimized care practices in pediatric emergency services.

However, the study has several limitations. First, the research was conducted in a single hospital setting, which may compromise the generalizability of the findings. Future studies should involve larger sample sizes or adopt a multicenter research design to validate the conclusions. Second, the current study lacks qualitative feedback from patients and their families. To gain a more comprehensive understanding of patient and family needs, preferences, and satisfaction levels, future research should incorporate qualitative methodologies, such as in-depth interviews or focus groups. Third, potential biases may exist in the data sourced from the HIS due to incomplete or inaccurate data records. Researchers should exercise greater caution in data cleaning and verification or explore alternative data collection methods to mitigate such issues in subsequent studies. Additionally, there may exist potential confounders, such as changes in hospital protocols or variations in clinical practices during the research period, which may have influenced the observed outcomes. Future studies should adopt more rigorous research designs, such as propensity score matching, to account for the confounders. Last, the lack of blinding in the study design may introduce observer bias. Future studies should prioritize blinding procedures wherever feasible. Overall, these limitations highlight important directions for future research to enhance the validity and generalizability of the findings in this study.

## Conclusion

In a word, this study confirms that optimized pediatric emergency care measures are effective in increasing successful resuscitation, improving recovery, reducing adverse events and increasing family satisfaction. These findings underscore the clinical viability of these interventions, we believe the optimized pediatric emergency care measures can be adopted as a valuable and scalable approach for integration into pediatric emergency practice across diverse healthcare settings.

## Data Availability

The raw data supporting the conclusions of this article will be made available by the authors, without undue reservation.
